# Components of the dorsal-ventral pathway also contribute to anterior-posterior patterning in honeybee embryos (*Apis mellifera*)

**DOI:** 10.1186/2041-9139-5-11

**Published:** 2014-03-12

**Authors:** Megan J Wilson, Nathan J Kenny, Peter K Dearden

**Affiliations:** 1 Laboratory for Evolution and Development, Genetics Otago and Gravida, Department of Biochemistry, University of Otago, P.O. Box 56, Dunedin 9054, New Zealand; 2 Developmental Biology Laboratory, Department of Anatomy, University of Otago, P.O. Box 56, Dunedin 9054, New Zealand; 3 Current address: Evolution and Development Research Group, Department of Zoology, University of Oxford, South Parks Road, Oxford OX1 3PS, UK

**Keywords:** Anterior posterior, *Apis mellifera*, Axis formation, Dorsal ventral, Dpp, Evolution, Honeybee, Toll

## Abstract

**Background:**

A key early step in embryogenesis is the establishment of the major body axes; the dorsal-ventral (DV) and anterior-posterior (AP) axes. Determination of these axes in some insects requires the function of different sets of signalling pathways for each axis. Patterning across the DV axis requires interaction between the Toll and Dpp/TGF-β pathways, whereas patterning across the AP axis requires gradients of bicoid/orthodenticle proteins and the actions of a hierarchy of gene transcription factors. We examined the expression and function of Toll and Dpp signalling during honeybee embryogenesis to assess to the role of these genes in DV patterning.

**Results:**

Pathway components that are required for dorsal specification in *Drosophila* are expressed in an AP-restricted pattern in the honeybee embryo, including Dpp and its receptor Tkv. Components of the Toll pathway are expressed in a more conserved pattern along the ventral axis of the embryo. Late-stage embryos from RNA interference (RNAi) knockdown of Toll and Dpp pathways had both DV and AP patterning defects, confirmed by staining with *Am-sna, Am-zen, Am-eve*, and *Am-twi* at earlier stages. We also identified two orthologues of *dorsal* in the honeybee genome, with one being expressed during embryogenesis and having a minor role in axis patterning, as determined by RNAi and the other expressed during oogenesis.

**Conclusions:**

We found that early acting pathways (Toll and Dpp) are involved not only in DV patterning but also AP patterning in honeybee embryogenesis. Changes to the expression patterns and function of these genes may reflect evolutionary changes in the placement of the extra-embryonic membranes during embryogenesis with respect to the AP and DV axes.

## Background

A critical early step in establishing the future body plan of any animal is the formation of the major body axes, the anterior-posterior axis (AP), and the dorsal-ventral axis (DV). Determination of these axes is established by an initial, often maternally driven, symmetry-breaking event, and as the process continues it requires precise zygotic control of gene expression across the body axes via cell-signalling pathways. DV patterning has been well studied in *Drosophila melanogaster*, which has a derived mode of development compared to other insects [1,2]. One unique innovation present in the DV axis patterning mechanism of Cyclorraphan flies such as *Drosophila* is the fusion of the extraembryonic membranes, which arise as a single tissue, the amnioserosa [3]. In the majority of insects, including other Dipterans, these tissues arise separately [2-6]. A conserved requirement in serosa patterning in many insects is the expression of *zenknüllt* (*zen*), a *Hox3* gene that has been co-opted, during insect evolution, into defining the serosal membrane [3,4,7,8]. In some insects, such as the honeybee, this expression also delineates where the amnion develops, and it has been suggested that it defines the border between the extraembryonic tissue and embryo [4].

Patterning of the dorsal epidermis and refinement of *zen* expression in *Drosophila* embryos, requires the expression of *decapentaplegic* (*Dpp*) at the dorsal midline. *Dpp* is a member of the TGF-β family of signalling molecules, which activate the MAD pathway on the dorsal midline of the blastoderm embryo in a gradient-like fashion. Short gastrulation (Sog) acts by binding to and inhibiting Dpp activity, thus promoting formation of the presumptive neuroectoderm. In dorsal regions, Tolloid (a dorsally restricted protein) cleaves Sog to release Dpp, enhancing its activity [9]. This limits Dpp activity to the dorsal side of the embryo, where high levels of signalling leads to up-regulation of genes required for the specification of dorsal ectoderm epidermis and amnioserosal fate [10-12], and allows, in ventral regions with low Dpp activity, neuroectoderm to form. *Megaselia abdita,* a basal Cyclorraphan fly, also requires *Dpp* and *zen* expression for development of distinct amnion and serosa tissues [3,5]. In the beetle *Tribolium castaneum, Dpp* expression mirrors where *zen-1* mRNA is found, and where the serosa-embryo border is positioned [13], indicating that Dpp has a conserved role in DV patterning. Using the honeybee (*Apis mellifera*) model, we previously found that *Dpp* mRNA was maternally expressed and localized to the dorsal side of the oocyte, rather than the embryo, and the MAD pathway was activated in overlying follicle cells, implying that the Dpp-MAD pathway may pattern the DV axis of the oocyte, rather than the embryo [14].

DV axis formation is initiated in the oocyte prior to fertilization and oviposition long before localized expression of Dpp in *D. melanogaster*. The first symmetry-breaking event is movement of the oocyte nucleus to an anterior-dorsal location [15]. This results in the asymmetric localization of *gurken* mRNA, encoding a TGF-β-like ligand, which in turn activates the EGF receptor in the overlying follicle cells [16]. Expression of *pipe* (*pip*) is restricted to ventral follicle cells, triggering a ventral protease cascade that cleaves and activates the signalling molecule Spätzle (Spz) [17]. Shortly after egg laying, active Spz protein binds to the Toll receptor on the ventral side of the egg resulting in localized ventral activation of the Toll pathway [18]. While there is no gene encoding an orthologue of *gurken* in most other insect genomes [19-21], there is another TGF-β-like member that is predicted to perform a similar role in DV patterning. TGF-β-like mRNAs are expressed maternally in *Tribolium, Nasonia*, and honeybee, but only localized to the anterior-dorsal side of the oocyte in a similar manner to *gurken* mRNA in *Nasonia* and the honeybee [14,21].

A key target of the Toll pathway is Cactus. Phosphorylation of Cactus results in its degradation on the ventral side of the embryo [22,23]. Cactus is an inhibitor of Dorsal, a DNA-binding rel/NF-κB protein family regulated by nuclear-cytoplasmic shuttling [24]. Cactus normally binds to Dorsal and sequesters it in the cytoplasm. As Cactus is degraded, however, on the ventral side of the embryo, Dorsal is free to translocate to the nucleus to regulate the expression of genes required for mesoderm and neuroectoderm patterning. In both short and long germ-band insects, loss of Toll signalling has been observed to result in dorsalization of the embryo and loss of the Dorsal DV gradient [25]. There are some subtle differences in the DV patterning network between *Tribolium* and *Drosophila* as, in *Tribolium*, Tc-Dorsal activates transcription of *Tc-cact* to negatively regulate its own function [25].

There is also emerging evidence for ‘cross-talk’ between AP and DV pathways in some insect groups, coordinating positional information across the body axes*.* The homeodomain-containing gene *orthodenticle* (*otd*) is required for anterior patterning in long and short germ-band arthropods [7,26-28]. DV fates are organized along the AP axis in a short germ-band insect *Tribolium* and this requires *Tc-otd*[29]*. Otd*, in honeybee embryos, controls formation of the serosa via regulation of *Am-zen* expression [7]. Additionally, the anterior patterning gene *hunchback* (*hb*) specifies correct *zen* expression in dorsal regions of the honeybee embryo [7].

These differences in the regulatory networks that define DV fates, combined with the relatively conserved nature of the molecules involved, make DV patterning an interesting model system in which to study the evolution of developmental pathways. To further understand how this pathway has changed over the course of insect evolution we examined the expression of some of the key orthologues of DV patterning genes during development of the honeybee embryo. Honeybee embryos follow a long germ-band mode of development that may have evolved independently from long germ-band development in *Drosophila*[30]. Using RNAi knockdown and *in situ* hybridization, we demonstrate that, while patterning of the ventral neuroectoderm via *snail* and *twist* expression and their necessity in refining *zen* expression is conserved, early-acting components of DV patterning are expressed differently to other insects, and RNAi knockdown of those components results in defects across both AP and DV axes.

## Methods

### Phylogenetics

Dorsal homologues were identified by tBlastN searches of insect genomes [31]. Multiple alignments of putative dorsal proteins were carried out in ClustalX. The resulting multiple alignment was analyzed in MrBayes 3.1.2 under the WAG model with default priors [32]. The Monte Carlo Markov Chain search was run over 1,000,000 generations with trees sampled every 1,000 generations. The first 250,000 generations were discarded as ‘burnin’. The final tree was displayed in Dendroscope [33].

### *In situ* hybridization

PCR-amplified cDNA fragments for all genes of interest (primer sequences for each gene are given in Table 1) were cloned into the pBluescript vector using standard cloning methods [13] for use as *in situ* hybridization probes. Digoxygenin (DIG)-RNA probes were synthesized and used in *in situ* hybridization experiments as described previously [30,34]. *Am-Dpp* RNA probe cloning has been described elsewhere [14]. DAPI staining was performed as previously described [35].

**Table 1:** List of oligonucleotide primers used in this study

**Target**	**F primer**	**R primer**
*Am-Twi*	CAGCTCTGGAATTCCTCATAGTG	CCACCTGCTTGTATCGCTG
*Am-sog*	GCAGAATGTACGTTCGGCA	GGCACGATAACGAGATAATTGTC
*Am-spz*	CGCAATTTGCCAGTTCCA	CTATCAGATTCAAAATCGATGTGAC
*Am-pipe*	GAAGTTCGCCTTCAACCTG	CACGGTCCGCCTTAAGTACA
*Am-GB18032*	CGAAAGACAAACCATACAGACC	ATGTGTACCGGGACTGGCT
*Am-sim*	CTTTGGACGGTTTCGTGTTT	GGTCAATTGTGACACGTTCG
*Am-NK2.2*	ACGATGTTCCGTGACATAC	CTCTCTTCGTCTTGTACCTGTG
*Am-GB19066*	TCTTCCGCCAATCAAAATTC	CTCTTTAACGGCGAGGACAG
*Am-Toll*	ATTCCATTCGTCCCCAAAC	CATTATTTGATAATAGTAACT
*Am-sna*	TTCACCACCATCACCATTACC	CGGAATACGTGGAGTACGATTT
*Am-cactus*	CACCTTTACACTTGGCTGTATTG	TCAGGAAGTGGTTCTGGTATTG
*Am-pnr*	GAAGGAGACGTTGACGAAGC	CGCCACTGGATTGGTTAGTT
*Am-tkv*	AAATACGGCCTGTGGATCAG	CACTTTTTCGCCTCTCCATC
*Am-tld*	AGAACGCTTATCGTGGCAAT	ACTGCTAGCACCCATGCTCT

### Honeybee RNA interference (RNAi)

Targeted mRNA knockdown was performed as described by Wilson and Dearden [36,37]. Briefly, dsRNA was synthesized from cDNA fragments of *Am-Toll*, *Am-Dpp*, and *Am-GB19066* cloned into pLitmus38i (NEB) using the MEGAscript RNA kit (Ambion). dsRNA was injected at 2.5 mg/mL in reverse osmosis-purified H_2_0 into freshly laid honeybee embryos. For each target, between 300–500 embryos were injected, with 15–20% surviving the injection process. Injected embryos were incubated for 30 h (stage 4) or 48 h (stage 9) at 35°C (80% humidity) before collection for *in situ* hybridization or DAPI staining.

## Results and discussion

### Expression of early-acting dorsal-ventral components in honeybee embryos

**Figure 1:**
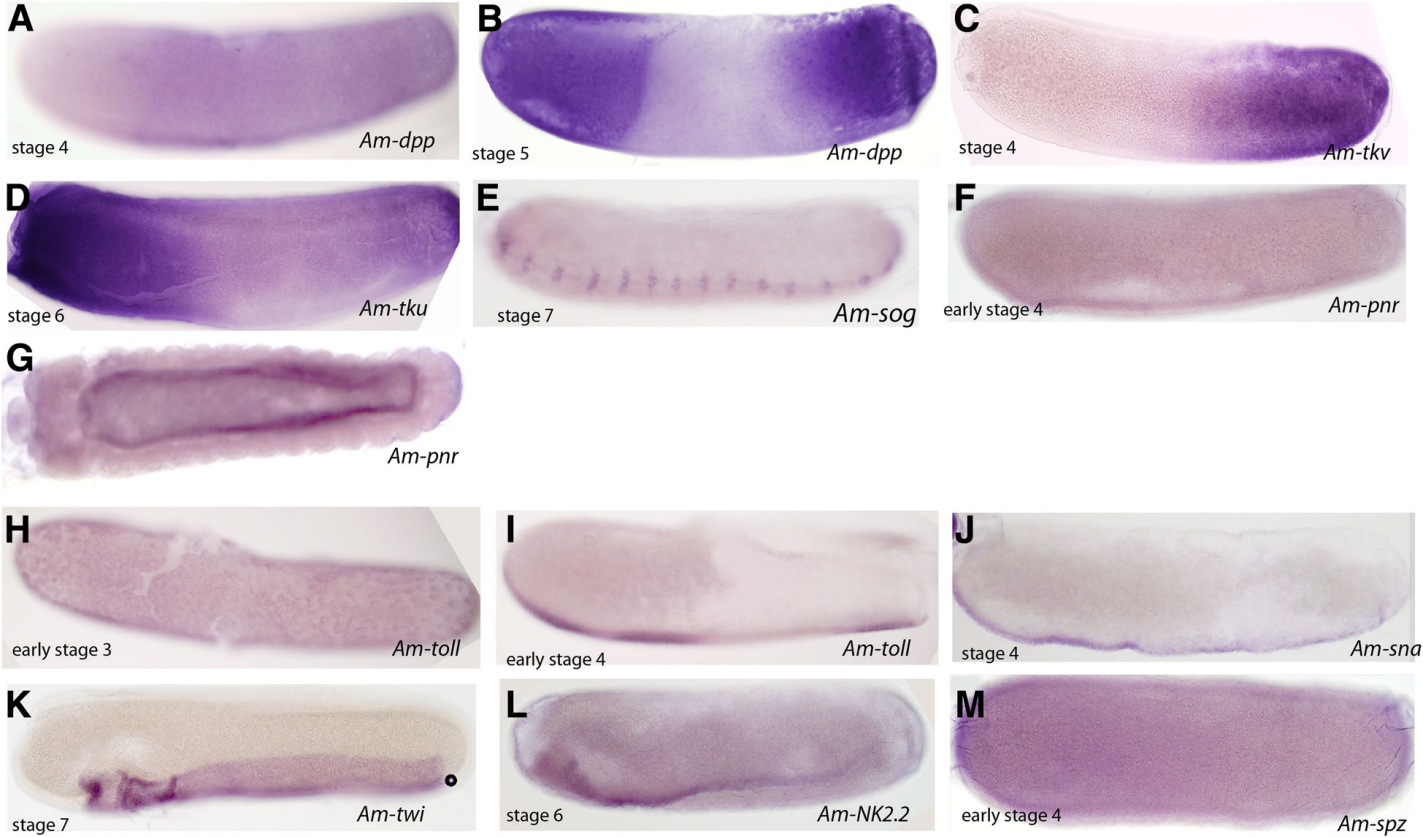
**Early expression of *Apis *genes likely to be involved in DV patterning. (A)***Am-Dpp* is expressed weakly through the posterior two-thirds of a stage 4 embryo. **(B)** By stage 4 *Am-Dpp* RNA expression is found at the anterior and posterior pole and absent from the central region of the embryo. **(C)***Am-tkv* mRNA is found at throughout the posterior half of the embryo. **(D)** After commencement of gastrulation, *Am-tkv* mRNA is strongly upregulated in an anterior domain and a weaker posterior terminal domain. **(E)** Expression of *Am-sog* is first detected at stage 7 on the ventral side of the embryo in a segmented pattern corresponding to development of the nervous system. **(F)** RNA for *Am-pnr* is weakly detected in young embryos and was not strongly detected until stage 9, in cells lining the yolk cavity **(G). (H)***Am-Toll* mRNA is detected throughout the early stage 3 embryo in a pattern suggesting that it is in association with energids. **(I)** By stage 4, *Am-Toll* is expressed in distinct regions along the ventral surface of the embryo. **(J)** mRNA for *Am-sna* and *Am-twi***(K)** are detected through the ventral ectoderm, where the prospective neuroectoderm will form. **(L)***AmNk2.2* mRNA is expressed in a layer of cells located at ventral ectoderm folds during gastrulation. These cells will later fuse at the mid-line to contribute to the neuroectoderm. **(M)***Am-spz* is expressed throughout the developing embryo (stage 4 shown). All embryo images are oriented with anterior to the left and dorsal side up.

We examined the expression patterns of honeybee orthologues of components of the *Drosophila* dorsal-ventral pathway. The BMP/Dpp pathway is a cascade, conserved in evolution, involved in DV patterning in both invertebrates and vertebrates [38]. We have previously shown that *Am-Dpp* is expressed maternally and that its mRNA is localized to dorsal regions of the oocyte [14], implying a possible early role in DV patterning during oogenesis. During embryogenesis, *Am-Dpp* mRNA is restricted to cells in the posterior two-thirds of the embryo prior to gastrulation (Figure 1A, stage 4) and is then upregulated as two distinct anterior and posterior domains of cells by stage 5 (Figure 1B).

This expression pattern is unusual when compared with *Dpp* expression in the Diptera. In *Drosophila,* zygotic *Dm-Dpp* mRNA is localised to dorsal regions of the syncytial blastoderm embryo and has a role in patterning the dorsal ectoderm and amnioserosa [11,39]. Two other members of the Dipteran suborder Brachycera, the scuttle fly (*Megaselia abdita*) and hoverfly (*Episyrphus balteatus*), have similar dorsal expression domains of *Dpp*[5,40]. The Nematocerans *Clogmia albipunctata* (moth midge) and *Anopholes gambiae (*mosquito) have a different pattern of *Dpp* expression; *Ca-Dpp* is expressed at the anterior and posterior ends of the blastoderm embryo [38], while *Ag-Dpp* mRNA is initially expressed as a broad medio-lateral band extending from the anterior to the posterior of the embryo corresponding to the presumptive amnion, but absent from the dorsal midline region where the presumptive serosa is patterned (as determined by *Ag-zen* expression) [41]. *Tribolium Dpp* (*Tc-Dpp*) is initially expressed throughout the blastoderm embryo, with higher expression at the anterior pole. *Tc-Dpp* expression at the anterior pole is later lost and *Tc-Dpp* mRNA is detected at the border between serosa and germ rudiment in a stripe along the AP axis [13]. In the locust *Schistocerca gregaria, Sg-Dpp* RNA is detected at the posterior of the germ band and head lobes at 15% development as well as in necklace cells that include presumptive serosa cells [42]. These differences in the expression of Dpp seem to correlate with where the extra-embryonic membranes are positioned, particularly the way the serosa is determined, and whether it grows to surround the entire embryo or just a portion of the embryo.

To further understand the unusual observed expression pattern of *Am-Dpp*, we also examined the expression of a Dpp receptor, *thickveins* (*Am-tkv*), in honeybee embryos. Knockdown of both maternal and zygotic *tkv* in *Drosophila* produces a phenotype identical to loss of *Dpp*[43]. *Am-tkv* mRNA is detected initially throughout the posterior two-thirds of the blastoderm embryo (Figure 1C), followed by expression in the anterior and posterior domains of cells at stage 5 (Figure 1D), and is expressed in a similar pattern to that of *Am-Dpp* (Figure 1B). In contrast, in the dipteran *Drosophila* and *Megaselia*, *tkv* is initially ubiquitous and then becomes restricted to the dorsal side of the embryo, head mesoderm, and ventral ectoderm during cellularization [44]. *A. gambiae tkv* mRNA is expressed in an anterior cap of cells and dorsal-posterior end of the embryo and appears absent from the presumptive serosa [41]. This suggests that the expression pattern of the *Tkv* receptor may have co-evolved with regulation of Dpp expression.

Sog acts as an antagonist to the BMP/Dpp pathway by binding to, and trafficking, the Dpp protein [45]. *Apis sog* mRNA, however, is not detected in early embryos; instead, expression of *Am-sog* is first detected after gastrulation, in short stripes of cells across the ventral surface in presumptive neurectoderm (Figure 1E). In contrast, *Tribolium sog* mRNA, which is expressed in the blastoderm in a ventral stripe on the embryo, acts to control Dpp movement [46]. In *Drosophila*, sog has an essential transport role that shapes the Dpp gradient; while more ventrolaterally, Dpp inhibition is augmented by a transcriptional repressor, Brinker, to promote ventral neuroectoderm development [47]. No orthologue of *sog* has been identified in *Nasonia* genome or transcriptome sequences [48]; however, potential orthologues of *brinker* are found in both honeybee and *Nasonia* genomes (accession numbers XP_003249310.1 and NV24459-PA respectively; unpublished data).

*Pannier* mRNA, encoding a zinc finger transcription factor, is upregulated in dorsal regions of the embryo by Dpp signalling in *Drosophila. Am-pnr* is not expressed during early to mid-stages of embryogenesis (Figure 1F), and it is not until the late stages that expression of *Am-pnr* is detected on ventral edges of the amnion (Figure 1G).

*Am-Toll* mRNA is expressed maternally and is found in association with energids throughout stage 1 embryos (Figure 1H). Just prior to gastrulation, *Am-Toll* mRNA becomes restricted to cells at the ventral surface of the embryo (Figure 1I), a pattern typical of the expression of *Toll* orthologues. We also examined the expression of factors that act downstream of the Toll pathway in *Drosophila* to pattern the mesoderm and neuroectoderm – *Am-sna*, *Am-twi*, and *Am-Nk2.2.* These mRNAs are detected in cells on the ventral side of the embryo (Figure 1J-L) and later in the nervous system (data not shown) a typical expression pattern for these genes. The *Apis* Toll ligand *spätzle* (*Am-spz*) mRNA is detected throughout honeybee embryonic ectoderm (Figure 1M). This differs from *Drosophila,* where maternal *spz* transcripts are found throughout the syncytial blastoderm, but no zygotic expression of *Dm-spz* is detected until late stages in the gut [18].

**Figure 2:**
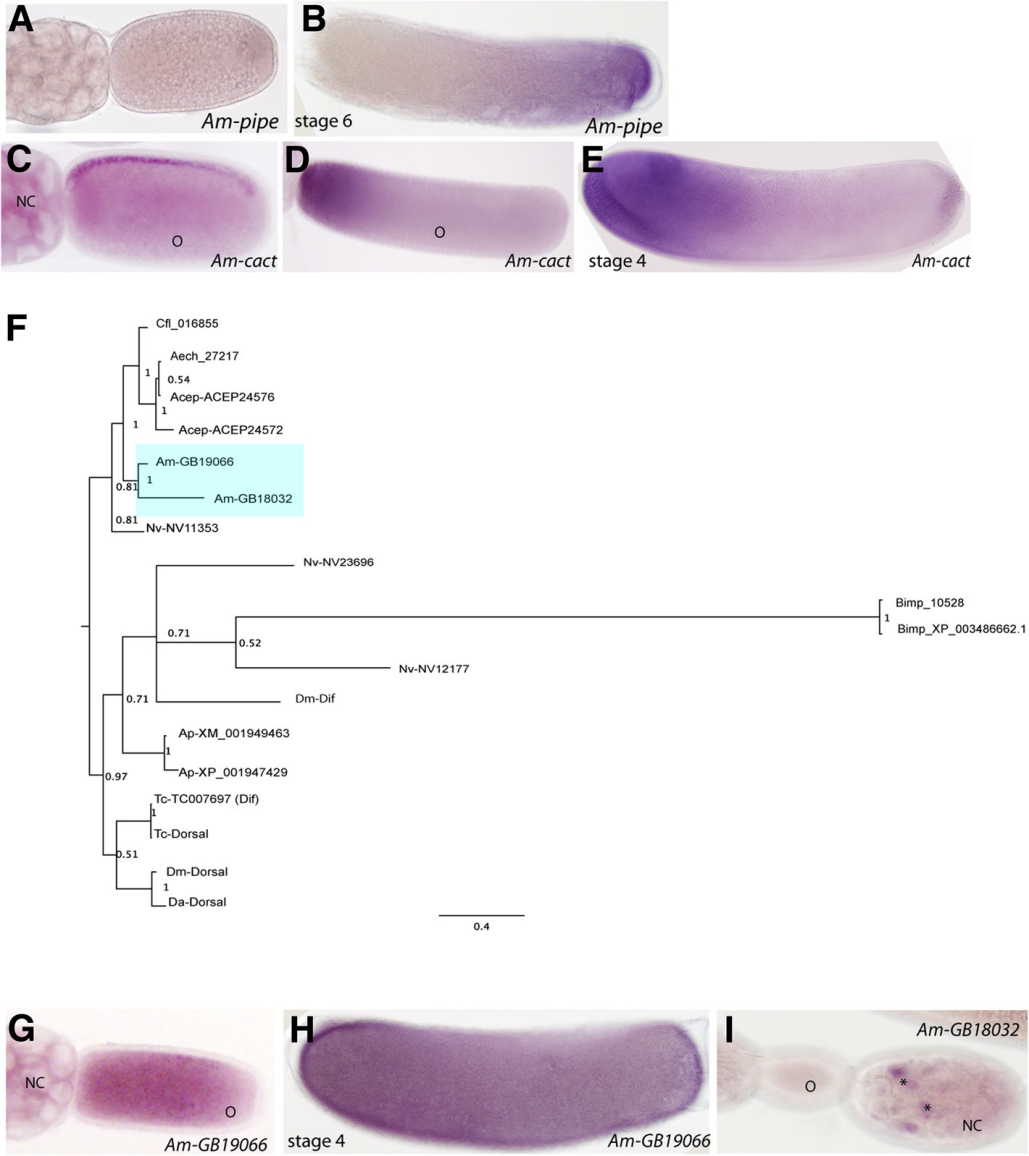
**Phylogenetic analyses of insect Dorsal-like proteins and expression of up-stream factors. (A)** No expression of *Am-pipe* mRNA is found in honeybee ovarioles. **(B)***Am-pipe* mRNA is first detected throughout the posterior third of the stage 6 embryo **(C)***Am-cact* mRNA is enriched to the dorsal side of the oocyte (stage 4 oocyte shown). **(D)** By the end of oogenesis (stage 9), *Am-cact* mRNA is enriched to the anterior pole of the oocyte. **(E)** Prior to gastrulation, *Am-cact* mRNA expression remains restricted to an anterior domain in the cellularised embryo. **(F)** Consensus Bayesian analysis tree for insect Dorsal-like proteins. Honeybee Dorsal-like proteins are indicated by shading. Numbers shown at the nodes are posterior probability values. **(G)***Am-GB19066* RNA is detected in the nurse cells and throughout the oocyte (mid-stage oocyte shown). **(H)***Am-GB19066* RNA is found throughout stage 4 embryos. **(I)** Expression of *Am-GB18032* RNA is only detected in the posterior nurse cells during mid-oogenesis (asterisks). Abbreviations: *Apis mellifera* (*Am*), *Drosophila melanogaster* (*Dm*), *Tribolium castaneum* (*Tc*), *Bombus impatiens* (*Bimp*), *Nasonia vitripennis* (*Nv*), *Atta cephalotes* (*Acep*), *Acromyrmex echinatior* (*Aech*), *Camponotus floridanus* (*Cf*), *Bombyx mori* (*Bm*), *Delia antiqua* (*Da*), *Acyrthosiphon pisum* (*Ap*), Nurse cell (NC), oocyte (o). All embryo images are oriented with anterior to the left and dorsal side up.

A key component of the ventral pathway is Pipe. Restriction of *Dm-Pipe* to the ventral follicle cells in *Drosophila* egg chambers results in local activation of proteinases required for activation of the Toll ligand, spz. *Apis Pipe* RNA is not expressed during oogenesis (Figure 2A), and not detected until stage 6 in the posterior half of the embryo (Figure 2B). This expression pattern is different to that observed in *Drosophila*, where *Dm-Pipe* is maternally provided and becomes restricted to the ventral side of the oocyte (in follicle cells) [49].

Nuclear entry of Dorsal protein is regulated by Cactus, a IκB homolog, in both *Drosophila* and *Tribolium*[24,25]. *Am-cact* RNA is expressed maternally (Figure 2C,D), becoming enriched to the dorsal side of the oocyte during mid-oogenesis (Figure 2C) and then towards the anterior pole just before fertilization and laying (Figure 2D). In blastoderm-stage honeybee embryos, *Am-cact* RNA is present in the anterior half, with weak staining at the posterior pole (Figure 2E). This embryonic expression pattern is unusual; in *Drosophila* and *Tribolium*, *Cactus* is expressed only in a ventral stripe in the blastoderm embryo, and only the maternal contribution of Cactus has been ascribed a function [25]. In *Nasonia*, however, *Nv-cact* is initially expressed as a ventral stripe but prior to gastrulation only a terminal ‘spot’ of expression remains [48].

BLAST searches revealed the presence of two possible Dorsal orthologues in the honeybee genome. Rel/NF-κB proteins (such as Dorsal) all contain a rel homology domain required for DNA binding and dimerization [50]. They can be split into two classes: Class 1 includes the Relish proteins that have ankryin repeats at the C-terminus, while Class 2 includes Dorsal-like proteins that have a transactivation domain and act as transcriptional activators. We carried out phylogenetic analysis of the Dorsal/rel group of proteins from insects, including Dorsal and Dorsal-related immunity factor (Dif) proteins. Dipteran and coleopteran genomes encode two Dorsal-like proteins (Figure 2F), while hymenopteran (*Nasonia*, *Apis mellifera*, *Bombus*, and *A. cephalotes* (leaf cutter ant)) genomes contain between two to three Dorsal-like genes (Figure 2F).

**Figure 3:**
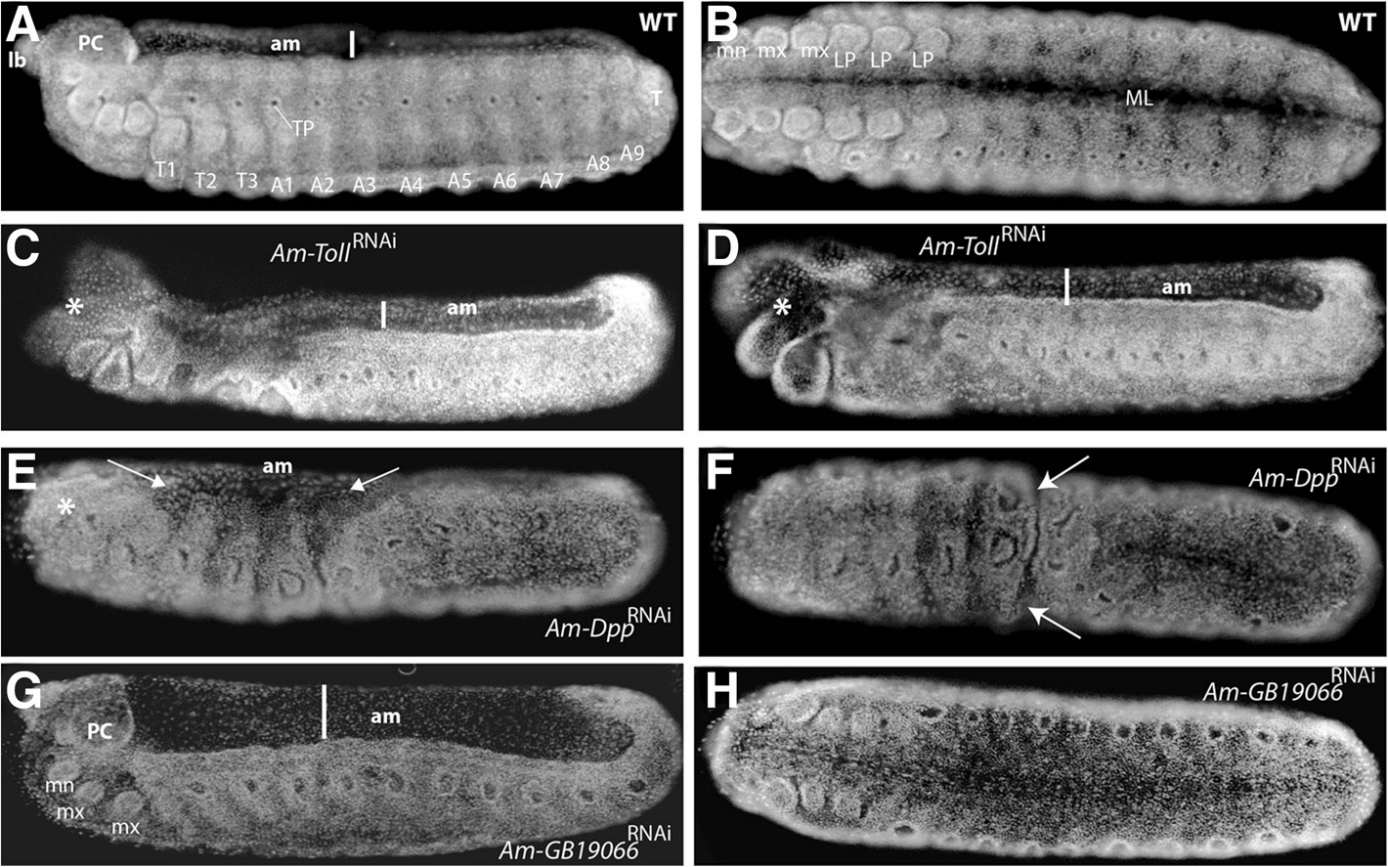
**Embryos resulting from dsRNA-mediated knockdown of *Am-Toll*, *Am-Dpp*, and *Am-GB19066*. (A)** DAPI stained wildtype (WT) embryo (stage 9, 48 h) with segments and appendages labelled. **(B)** Ventral view of a WT embryo at stage 9. **(C)** Stage 9 embryo following injection with dsRNA against *Am-Toll*. **(D)** A second example of typical stage 9 embryo after knockdown of *Am-Toll*, **(E)***Am-Dpp*^RNAi^, and **(F)***Am-Dpp* dsRNA knockdown ventral side up. **(G)** and **(H**, ventral view**)***Am-GB19066*^RNAi^. All embryo images are oriented with anterior to the left and dorsal side up unless stated otherwise. Abbreviations: midline (ML), amnion (am), procephalic lobe (PC), mandible (mn), maxillae (mx), limb pair (lp), thorax (T), abdominal (A), terminal segment (T), tracheal pit (TP).

Since neither Dorsal-like honeybee protein could be easily distinguished as being more ‘Dorsal-like’ based solely on sequence (Figure 2F), we examined the maternal and embryonic expression of both *Apis Dorsal-like* genes, *GB19066* and *GB18032. Am-GB19066* mRNA is maternally expressed and detected throughout the oocyte from mid-oogenesis (Figure 2G). During embryogenesis, expression is detected throughout the ectoderm of the developing embryo with enrichment at the ventral and AP termini (Figure 2H, stage 4). *Am-GB18032* expression could only be detected in a subset of nurse cells at the posterior end of the nurse cell cluster in queen ovarioles (Figure 2I). No expression of *Am-GB18032* was detected in oocytes or embryos (Figure 2I, data not shown). Since *Drosophila* and *Tribolium Dorsal* mRNAs are expressed throughout the embryo [51], we proposed *Am-GB19066* as the most likely functional orthologue of Dorsal in honeybee, and undertook further analysis of this gene by dsRNA knockdown (Figure 3G,H).

### RNAi knockdown of *Am-Toll*, *Am-GB19066 (Dorsal)*, and *Am-Dpp*

Despite widespread conservation of *Drosophila* DV patterning genes between insect orders, the *Apis* orthologues of these genes have divergent patterns of expression compared to other insects, particularly Diptera. We examined the function of three key DV genes by dsRNA knockdown, and assessed the phenotypes produced in 48 hour embryos, a time at which morphological markers of DV patterning are present, using DAPI staining (Figure 3A,B, ventral view).

Targeted knockdown of *Am-Toll* by dsRNAi (*Am-Toll*^RNAi^) results in severe anterior defects and disrupted patterning along both AP and DV axes. Thoracic and anterior abdominal segments are fused and, while tracheal pits are visible, the segments are indistinguishable (indicated in Figures 3C and 1D). At the anterior end of the embryo the serosa membrane is retained (Figure 3C,D asterisks; nuclei are more sparsely distributed indicating serosa tissue), implying that the extraembryonic membranes have formed but, in the case of the serosa, have failed to expand to cover the embryo. Loss of function of *Tribolium Toll* results in the loss of DV asymmetry of the extra-embryonic tissue border, with retention of the amnion and serosa tissue [25].

**Figure 4:**
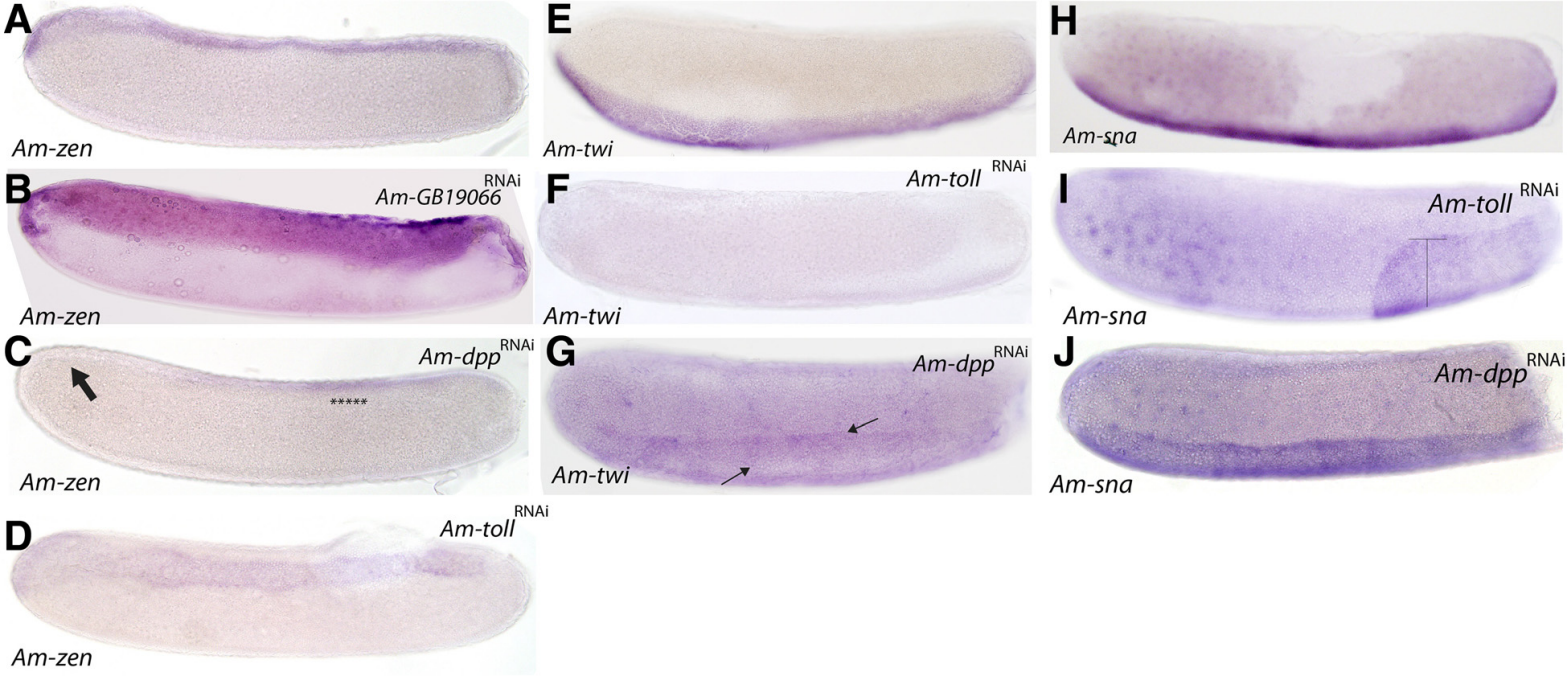
**Gene expression in *Am-Toll*, *Am-GB19066*, and *Am-Dpp *RNAi stage 4 embryos. (A)***Am-zen* stained control embryo, typical expression of *Am-zen* on the dorsal side of the embryo. **(B)** Knockdown of *Am-GB19066* results in expansion of *Am-zen* expression. **(C)***Am-Dpp*^RNAi^ treated embryos, loss of *Am-zen* expression from the anterior of the embryo some weak mid-to-posterior expression remains (asterisks). **(D)***Am-Toll*^RNAi^ embryo stained for *Am-zen* transcript results in mild expansion of *Am-zen* expression at the dorsal midline (indicated). **(E)** Control embryo stained for *Am-twi* expression. *Am-twi* is expressed along the ventral midline of the embryo. **(F)***Am-twi* expression is lost in *Am-Toll* knockdown embryos **(G)***Am-Dpp*^RNAi^ embryos stained for *Am-twi.* Arrows show centrally located bands of expression running along the anterior-posterior axis*.***(H)** Expression of *Am-sna* in control embryos. **(I)***Am-Toll*^RNAi^ embryos displayed loss of *Am-sna* expression from the anterior-ventral through to the posterior third of the embryo. The remaining posterior domain of *Am-sna* expression is expanded dorsally. **(J)***Am-Dpp*^RNAi^ embryos stained for *Am-sna*. Staining is detected as a slightly broader ventral expression domain (indicated) compared to controls. **(F-I)** Schematic representation of the AP and DV patterning defects in dsRNA-knockdown embryos. All embryos are stage 4 embryos, oriented with anterior to the left and dorsal side up.

*Am-Dpp*^RNAi^ stage 9 embryos exhibited loss of anterior appendages, with loss of overt signs of segmentation (Figure 3E,F) and the tracheal pits are also enlarged (Figure 4E). The amnion is reduced to cover a much smaller dorsal domain (arrows in Figure 3E). Loss of patterning is particularly obvious in the presumptive thoracic-anterior abdominal region (Figure 3E) and across the ventral side of the embryo there is a distinct groove (arrows Figure 3F) that appears to separate the two halves of the embryo.

*Am-GB19066* knockdown results in a considerable broadening of the amnionic tissue (Figure 3G). The head appendages are present and appear normal (Figure 3G,H). Thoracic abdominal and posterior tracheal pits are enlarged compared to wildtype (WT) embryos, and it is difficult to discriminate between body segments (compare Figure 3A to Figure 3G,H). These results imply that amnion patterning is disrupted with knockdown of a dorsal-like gene, with limited disorder of some ectoderm structures. This phenotype is milder than that of dorsal loss-of-function experiments in *Drosophila,* where mutants lack mesoderm and ventral ectoderm [52].

**Figure 5:**
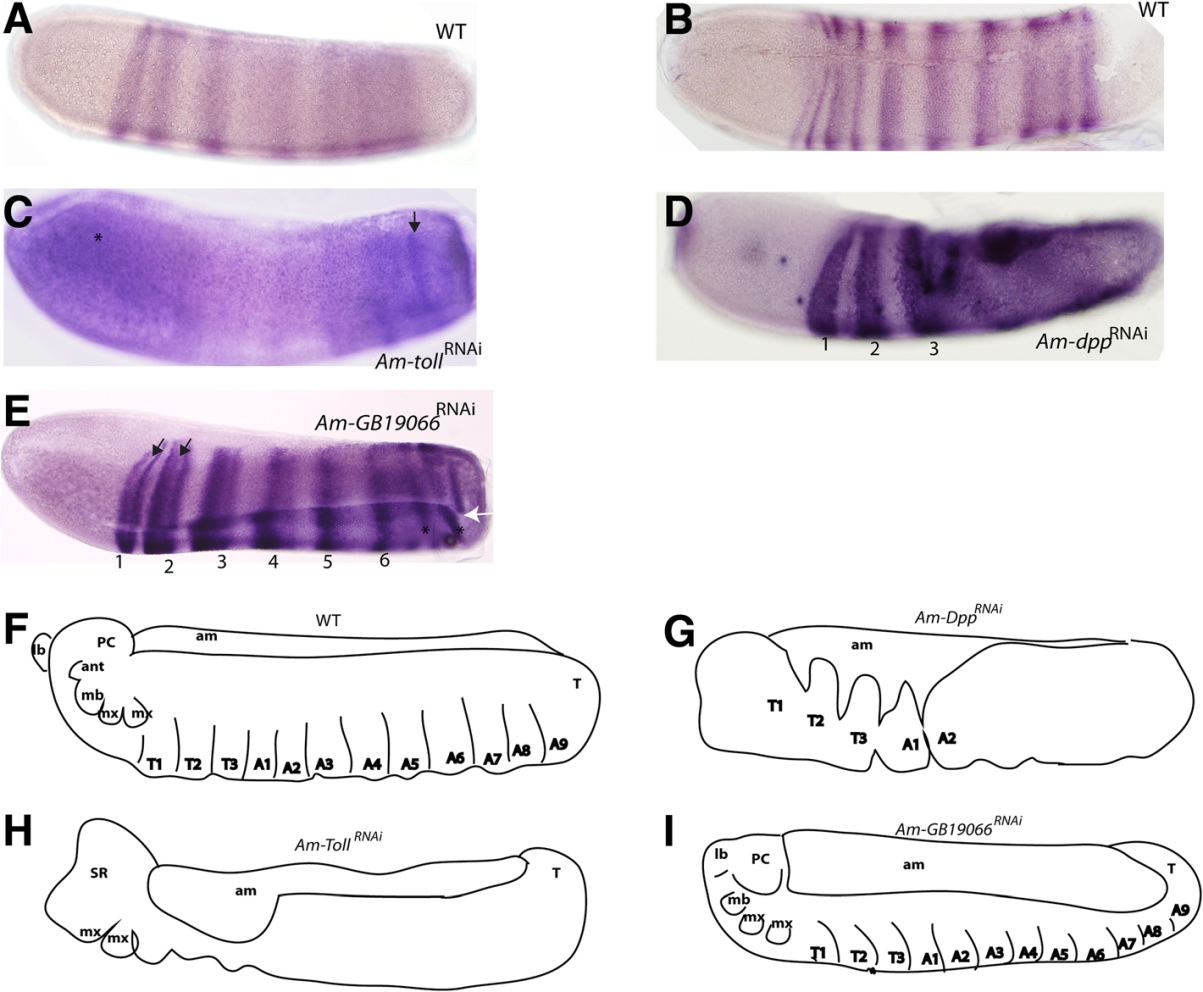
***Am-eve *expression in dsRNA knockdown embryos. (A** and **B)***Am-eve* expression at stages 3–4. *Am-eve* stripe expression starts at the anterior to posterior fashion. Stripes begin to split (arrow) and become distinguished just prior to gastrulation **(B). (C)***Am-eve* mRNA is detected throughout stage 4 embryos following knockdown of *Am-Toll*. The strongest expression is found at the anterior and posterior ends of the embryo, and some stripe expression can be discerned (arrow). **(D)** In *Am-Dpp*^RNAi^ embryos only the first three stripes (1–3) of *Am-eve* expression is seen. The posterior half of the embryo *Am-eve* mRNA appears as one broad domain of expression. **(E)***Am-eve* transcripts are expressed in stripes after *Am*-*GB19066*^RNAi^ but are much less distinct, particularly throughout the posterior half of the embryo. The gastrulation ventral furrow appears expanded and there is some disruption of *Am-eve* stripes across this region (white arrow). **F)** Schematic representation of WT embryos at stage 9 with segments labeled. **(G)** Representation of the *Am-Dp*p^RNAi^ phenotype showing loss of most segments and amnion. **(H)** Summary schematic of *Am-Toll*^RNAi^ embryos labeling remaining segmens present in these embryos. **(I)** Representation of *Am-GB19066*^RNAi^ labeling remaining segments present in RNAi treated embryos. Abbreviations: amnion (am), procephalic lobe (PC), mandible (mb), maxillae (mx), thorax segment (T1-3), abdominal segment (A1-9), terminal segment (T), antenna (ant), labrum (lb), serosa (SR).

We also examined the expression patterns of three key genes regulated by the Toll and Dpp pathways in *Drosophila* in honeybee dsRNA knockdown embryos. Zen acts to pattern the extraembryonic membranes in *Drosophila*, honeybee*,* and *Tribolium*[7,8,53]. In honeybee embryos, *Am-zen* is expressed in several cells along the dorsal midline of the embryo and in an anterior-dorsal cap (Figure 4A) [7]. In *GB-19066*^RNAi^ embryos, *Am-zen* expression expanded to cover almost a third of the dorsal side of the embryo (Figure 4B), suggesting that the dorsal-like protein GB19066 has a role in restricted Dpp expression to a few cells at the dorsal-midline of the embryo. *Am-Dpp*^RNAi^ resulted in loss of *Am-zen* expression from the anterior-dorsal region of the embryo (Figure 5C; the presumptive serosa [7]). *Am-zen* mRNA is still detected in a central to posterior-dorsal region of the *Am-Dpp*^RNAi^ embryo (Figure 4C, asterisks), typically where the amnion forms. Knockdown of *Am-Toll* resulted in an expansion of *Am-zen* expression across the dorsal midline (Figure 4E), in a domain almost double the number of cells of that seen in WT (Figure 4A).

The *twist* (*twi*) gene, an immediate downstream target of the Toll pathway, is required for mesoderm specification, being expressed as a ventral domain in most insects (Figure 4E) [48]. In *Tribolium*, *Tc-twi* is found expressed as a ventral domain located in the posterior growth zone [25]. *Am-twi* expression is completely lost in *Am-Toll*^RNAi^ embryos (Figure 4F). In *Am-Dpp* knockdown embryos, *Am-twi* mRNA is weakly detected thorough the entire ectoderm surface, although expression is stronger in a ventral domain (arrows, Figure 4G).

*Snail* expression is downstream of the Toll pathway and, as such, its expression in *Drosophila* and *Tribolium* is restricted to the ventral side of the embryo where it is required for gastrulation and neurogenesis [54-57]. This expression pattern is also conserved in honeybee embryos where *Am-sna* is expressed as a ventral stripe in blastoderm embryos (Figure 5H). In *Am-Toll*^RNAi^ embryos, ventral *Am-sna* expression is lost from the anterior and abdominal ventral surface; however, the remaining posterior expression has expanded dorsally to form a wider band of expression (Figure 5I). In *Am-Dpp*^RNAi^ embryos, *Am-sna* is also expressed as a ventral stripe, although this region is significantly expanded (Figure 5J).

Since many ‘DV pathway dsRNA’ treated embryos showed AP patterning or segmentation defects, we also examined the expression of *Am-even-skipped* (*Am-eve*). *Am-eve* mRNA in honeybee is maternally expressed, and in early embryos is detected throughout the syncytial blastoderm and early cellularized embryo [58]. It later becomes refined to a broad abdominal domain before distinct stripes appear. At stage 4, *Am-eve* is expressed as seven broad stripes that later split into 14 stripes to establish the parasegment boundaries during segmentation (Figure 5A,B) [59]. *Am-Toll*^RNAi^ results in disorganization of *Am-eve* expression at stage 4 (Figure 5C). *Am-eve* mRNA is found throughout the embryo with two possible distinct stripes found at the posterior end above the background of ubiquitous *Am-eve* expression (Figure 5C, arrows) and staining seems stronger at the anterior (Figure 5C, asterisk). Loss of *Am-Dpp* expression results in *Am-eve* expression throughout the mid-posterior end of the embryo, although the first three stripes are still distinguishable (Figure 5D). Following knockdown of *Am-GB1906*, *Am-eve* stripes 1–6 are distinct and normal in appearance, with stripes one and two beginning to split (Figure 5E, arrows). However the posterior end stripes appear fragmented and disorganized across the DV axis (Figure 5E, asterisks).

**Figure 6:**
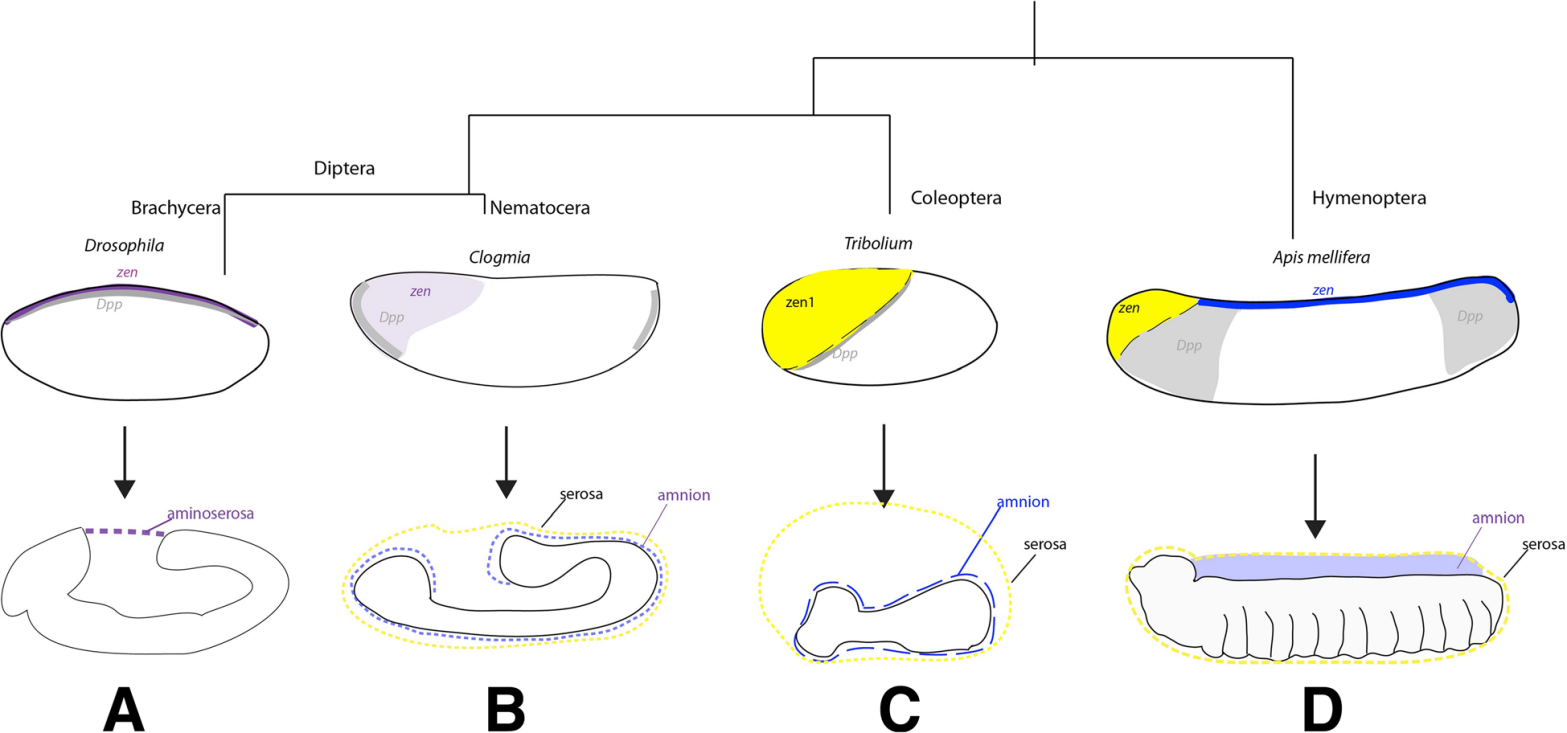
**Summary of extraembryonic membrane patterning across holometabolous insects with respect to *Dpp *and *zen *expression. (A)** In Cyclorrhaphan flies, such as *Drosophila,* the extraembryonic membranes develop as a single tissue, the amnioserosa (purple dashed), which is specified at the dorsal midline by *Dpp* (grey) and *zen* expression (purple). **(B)** In Dipteran Nematoceran flies, the serosa and amnion are specified separately by *Dpp* (grey) expression on the ventral side of either pole in the midge mite *Clogmia*[32] and *Ca-zen* expression (light purple) in an anterior-dorsal domain [60]. The serosa (yellow dashed line) developing to surround the entire germ band of the embryo [60]. **(C)***Tribolium zen1* expression (yellow) specifies the serosa only, *zen2* is required for fusion of the serosa and amnion following dorsal closure but not their specification [8]. *Tc-Dpp* (grey) is expressed across the DV axis at the serosa border [13]. In *Tribolium*, the serosa and amnion grow over the germ band to cover the embryo as separate surrounding membranes/cell layers. **(D)** In *Apis mellifera*, the amnion (pale purple) develops from dorsal midline (*zen* expressing cells, dark blue) to cover the yolk. The honeybee serosa tissue (yellow dashed line) is initially specified from cells at the anterior end of the blastoderm (which are also expressing *zen* (shown in yellow)) that move to surround the entire embryo. *Dpp* expression is found at the anterior and posterior ends of the embryo and is required for anterior-dorsal *zen* expression and serosa patterning.

*Am-Toll* and *Am-Dpp* have a greater influence on *Am-eve* expression (resulting in loss of the amnion and serosa tissues) than *Am-zen*, which has little effect on *Am-eve* stripe expression [7]. This implies the Dpp and Toll pathways influence the definition of the AP axis prior (Figure 5G,H) to establishment of *Am-eve* stripe expression during segmentation. This influence is in addition to their roles in DV axis establishment, and occurs independently of *Am-zen*.

## Conclusions

This study set out to describe the expression and function of the Toll, Dorsal, and Dpp pathways in the long germ-band insect, *A. mellifera,* during early embryo development. Comparisons of the expression and function of individual components of the DV patterning pathways in insects reveal the roles of some factors are more highly conserved, particularly those acting later in these pathways. This is consistent with evolutionary changes in segmentation and axis formation in insects, where earlier acting components are evolving more rapidly than downstream factors [7,27,36,61]. For example *sim*, *twist*, and *snail* are expressed along the DV axis of the long and short germ-band embryos and a *zen* gene is expressed in regions where extraembryonic membranes will form. The most striking differences in spatial expression are found during early development. In honeybee embryos, the expression of many factors, including *Am-Toll*, *Am-Dpp*, and *Am-cact*, appears to be oriented with respect to the AP axis of the early embryo. This may be partially due to the way in which the extraembryonic membranes are determined in different insects (Figure 6). Establishing the position of the serosa-embryo border differs, for example in *Tribolium* (Figure 6B), where the amnion-embryo border is positioned asymmetrically across the AP axis [25], while in the honeybee the serosa initially patterns an anterior cap before moving posteriorly to enclose the embryo (Figure 6D). In *Drosophila*, aminoserosa tissue originates at the dorsal midline following *zen* expression (Figure 6A). These changes to the positioning of the extraembryonic membrane borders appear to require shifts in the spatial and temporal expression of *Dpp* and *zen* as described in Figure 6.

Our data implies that signalling pathways have considerable influence over cell fate determination across both axes. Loss of honeybee Toll and Dpp function not only resulted in loss of DV patterning but also disruption to patterning across the entire ectoderm. This suggests that patterning of both major body axes may be more co-dependent than previous studies in *Drosophila* have led us to understand.

## Abbreviations

Am-eve: *Am-even-skipped*; Am-TollRNAi: *Am-Toll* by dsRNAi; AP: Anterior-posterior; DV: Dorsal-ventral; Dpp: *decapentaplegic*; hb: *hunchback*; otd: *orthodenticle*; pip: *pipe*; RNAi: RNA interference; Sog: Short gastrulation; spz: *spätzle*; Tc-Dpp: *Tribolium Dpp*; tkv: *thickveins*; twi: *twist*; WT: Wildtype; zen: *zenknüllt.*

## Competing interests

PKD is an editor of EvoDevo.

## Authors’ contributions

MJW conceived of the study, performed most of the experiments, and co-wrote the manuscript. PKD co-wrote the manuscript. NJK performed some of the cloning experiments and co-wrote the manuscript. All authors read and approved the final manuscript.
